# Nano-engineered microcapsules boost the treatment of persistent pain

**DOI:** 10.1080/10717544.2018.1431981

**Published:** 2018-01-31

**Authors:** Olga Kopach, Kayiu Zheng, Luo Dong, Andrei Sapelkin, Nana Voitenko, Gleb B. Sukhorukov, Dmitri A. Rusakov

**Affiliations:** a Department of Clinical and Experimental Epilepsy, UCL Institute of Neurology, University College London, London, UK;; b School of Engineering and Materials Science, Queen Mary University of London, London, UK;; c Centre for Condensed Matter and Materials Physics, Queen Mary University of London, London, UK;; d Department of Sensory Signaling, Bogomoletz Institute of Physiology, Kyiv, Ukraine

**Keywords:** Biodegradable microcapsules, persistent pain, Na^+^ channels, drug diffusion, neuronal excitability, pain relief, locomotive deficit and anxiety

## Abstract

Persistent pain remains a major health issue: common treatments relying on either repeated local injections or systemic drug administration are prone to concomitant side-effects. It is thought that an alternative could be a multifunctional cargo system to deliver medicine to the target site and release it over a prolonged time window. We nano-engineered microcapsules equipped with adjustable cargo release properties and encapsulated the sodium-channel blocker QX-314 using the layer-by-layer (LbL) technology. First, we employed single-cell electrophysiology to establish *in vitro* that microcapsule application can dampen neuronal excitability in a controlled fashion. Secondly, we used two-photon excitation imaging to monitor and adjust long-lasting release of encapsulated cargo in target tissue *in situ*. Finally, we explored an established peripheral inflammation model in rodents to find that a single local injection of QX-314-containing microcapsules could provide robust pain relief lasting for over a week. This was accompanied by a recovery of the locomotive deficit and the amelioration of anxiety in animals with persistent inflammation. *Post hoc* immunohistology confirmed biodegradation of microcapsules over a period of several weeks. The overall remedial effect lasted 10–20 times longer than that of a single focal drug injection. It depended on the QX-314 encapsulation levels, involved TRPV1-channel-dependent cell permeability of QX-314, and showed no detectable side-effects. Our data suggest that nano-engineered encapsulation provides local drug delivery suitable for prolonged pain relief, which could be highly advantageous compared to existing treatments.

## Introduction

Persistent or chronic pain remains largely resistant to treatment. In practice, the analgesics of choice have been opiates and Na^+^-channel blockers, such as lidocaine. However, their use has been restricted by side-effects, such as sedation for opiates and cardiotoxicity for lidocaine and its derivatives. These factors curtail the amount administered, mainly due to the risk of a rapid systemic escape from the target site or from a drug depot. Furthermore, existing methods of focal drug application have principal limitations: singular injections provide poor control over the application kinetics whereas chronic injecting devices are invasive, costly, and are prone to the infection risk.

Over the years, various medicine cargo systems have been explored and tested for their capability of targeted drug delivery (Sukhorukov et al., 2005; Tanbour et al., 2016), including pain treatment. Some notable attempts utilized hydrogels involving steroid treatment (Liu et al., 2016), photo-triggered liposomes carrying Na^+^-channel blocker tetrodotoxin (Rwei et al., 2015; Zhan et al., 2016), or immunoliposomes for opioid delivery (Hua & Cabot, 2013). A major challenge has been, however, to incorporate multiple functionalities into a single entity, which would deliver medicine to the target site and release it over a relatively long period, in a controllable fashion (Sukhorukov et al., 2005; Sukhorukov & Mohwald, 2007; Stuart et al., 2010). One promising direction has been biodegradable polyelectrolyte-based microcapsule systems constructed with the layer-by-layer (LbL) method (Decher, 1997; Stuart et al., 2010). Such systems could incorporate different polyelectrolytes and charged nanoparticles in a single-LbL capsule combining several functionalities. A wide variety of substances have successfully been tried as cargo for encapsulation (De Cock et al., 2010).

In brief, the LbL microcapsules could be made controllably to a size of ≥0.3 µm, with the outer layers providing their functional features, such as varied permeability (cargo release rate) or sensitivity to the external stimuli (pH, temperature, osmolarity, light, etc.) (Munoz Javier et al., 2008; Antipina & Sukhorukov, 2011; Delcea et al., 2011; Pavlov et al., 2011, 2013; Gao et al., 2016). The microcapsules are routinely made of biodegradable components and show no toxicity or appreciable inflammation or apoptotic effects when injected in tissue or internalized by cultured cells (Pavlov et al., 2011).

Among the analgesics used for local pain treatment there has been a growing interest in the Na^+^-channel blocker QX-314, which appears to produce relatively long-lasting analgesic effects, at relatively higher potency, in animal models (Lim et al., 2007; Roberson et al., 2011; Zhao et al., 2014). Whilst QX-314 is membrane-impermeable and blocks Na^+^ channels from the cytoplasm side, studies *in situ* have established that it enters nerve cells through the transient receptor potential (TRP) receptors TRPV1 and TRPA1 (Binshtok et al., 2007; Leffler et al., 2011; Stueber et al., 2016), which are strongly expressed in peripheral nociceptors (pain-signaling neurons), but also through a TRP-independent mechanism (Brenneis et al., 2014; Hofmann et al., 2014). These findings have prompted calls for further validation of this potentially efficient analgesic (Roberson et al., 2011; Stueber et al., 2016).

Notwithstanding the promise of LbL encapsulation technology, there have been no systematic attempts to adapt it to the explorative studies of therapeutic intervention pertinent to neuropathologies such as peripheral pain. We therefore set out to test whether drug delivery via nano-engineered LbL microcapsules has a therapeutic potential in the context of pain treatment. Here, we encapsulate QX-314 in biodegradable microcapsules made of poly-l-arginine (PArg) and dextran sulfate, adjust and test its release action *in vitro* and *in situ*, and document the effects of encapsulated QX-314 (injected subcutaneously) in a persistent inflammatory pain model in rodents *in vivo*.

## Materials and methods

### Design and fabrication of microcapsules

Polymer-based multilayer microcapsules were fabricated in accord with the previously established LbL assembly technique (Sukhorukov & Mohwald, 2007; Stuart et al., 2010). Quaternary lidocaine derivative QX-314 chloride, the membrane-impermeable blocker of voltage-gated Na^+^ channels, was encapsulated at variable amounts (4–10 pg/capsule). Briefly, CaCO_3_ was used as sacrificial templates, and PArg was deposited as the first layer and incubated for 15 min, then washed three times; the oppositely charged dextran sulfate sodium salt (DXS) was assembled as the second layer with the same procedure. In order to encapsulate QX-314 into the capsule shells, two of the DXS layers were substituted by QX-314 during the assembly process. Polymer solutions and QX-314 solutions were prepared at 2 mg/ml. For fluorescent visualization, one of the PArg layers was labeled with TRITC. After the assembly of eight layers, the CaCO3 cores were removed with 0.2 M EDTA. The resulting microcapsule architecture was PArg/DXS/QX-314/DXS/QX-314/DS/PArg-TRITC/DXS. Empty microcapsules (no payload) were used as control. The concentration of capsules in suspension was determined by a hemocytometer, Labtech International, Heathfield, UK (density range 1.1–2.2 × 10^8^ 1/ml). The QX-314 encapsulation rate was calculated by measuring the remaining QX-314 in supernatants. The suspension of microcapsules was stored at 4 °C. To assess the encapsulated cargo release rate, the microcapsules of a similar configuration were made, with one of the DXS layer substituted by Alexa Fluor 488 hydrazide (ThermoFisher Scientific, Paisley, UK).

### Primary neuron cultures

Hippocampal neurons were isolated from the Sprague-Dawley rat pups (P0–P2 day-old), in accordance with the European Commission Directive (86/609/EEC) and the United Kingdom Home Office (Scientific Procedures) Act (1986). Neurons were cultured in NeuroBasal A/B27-based medium on a rat astrocyte feeder layer at 37 °C as described (Ermolyuk et al., 2013). Cultured neurons were placed in a recording chamber mounted on the stage of an Olympus BX51WI upright microscope (Olympus, Tokyo, Japan) equipped with a LUMPlanFL/IR 40 × 0.8 objective coupled to an infrared DIC imaging system.

### Electrophysiology

Electrophysiological recordings were carried out using a Multipatch 700B amplifier controlled by the pClamp 10.2 software package (Molecular Devices, Silicon Valley, CA). Recordings were made in a bicarbonate-buffered solution (aCSF) containing (in mM) 126 NaCl, 3 KCl, 2 MgSO_4_, 2 CaCl_2_, 26 NaHCO_3_, 1.25 NaH_2_PO_4_, 10 d-glucose saturated with 95% O_2_ and 5% CO_2_ (pH 7.4; 300–310 mOsmol) at 31–33 °C. The recording electrode resistance was 2.5–5 MΩ when filled with the intracellular solution containing (in mM) 126 k-gluconate, 10 HEPES, 4 KCl, 4 MgCl_2_, 2 BAPTA, 4 Mg-ATP and 0.4 GTP-Na (pH 7.2 with KOH, osmolarity ∼290 mOsmol). For the intracellular delivery of microcapsules, the glass electrode tip was back-filled with a suspension of microcapsules supplemented to the intracellular solution. The presence of microcapsules at the pipette tip was confirmed by visualizing their TRITC (FITC) fluorescence (conjugated to the capsule shell) prior to the breaking into whole-cell mode. Once in whole-cell, neurons were monitored for changes in their intrinsic active and passive membrane properties using fast sampling (20 kHz) every 1–3 min, until the baseline had stabilized (40–50 min). The series of sub- and supra-threshold rectangular current pulses (500–1000 ms duration) of the gradually (stepwise) increased stimulus intensity were applied. The steady-state input resistance (R_input_) was estimated from the measured current–voltage relationship at various time points during the experiment. The maximal firing frequency and the corresponding (minimal) membrane voltage were monitored throughout. Changes in the action potentials (AP) shape were analyzed using the first AP spike responding to a slow-ramp input current (ramp slope 480 pA/s).

### Acute skin tissue preparation

Peripheral tissue was taken from the plantar surface of the hind paw of Sprague-Dawley rats (21 to 25-day-old) following animal sacrifice. Tissue sections (300–350 µm thickness) consisting of skin layers and the tissue beneath were cut using a tissue chopper (McIlwain Model TC752, Mickle Laboratory Engineering Co., Guilford, Surrey, UK). Acute slices were placed in the recording chamber mounted on the stage of an Olympus upright microscope (Olympus) and maintained in HEPES-based physiological buffer.

### Imaging of encapsulated drug release

Microcapsules containing Alexa Fluor-488 (similar molecular weight to QX-314) were delivered into acute skin tissue *in situ* with a glass pipette positioned between epidermal and dermal layers. One-photon (confocal) or two-photon (2P) excitation fluorescent imaging was carried out with either a Radiance 2000 (Zeiss-Bio-Rad, Jena, Germany) or a Femto2D (Femtonics, Budapest, Hungary) system optically linked to a Ti:Sapphire Mai-Tai femtosecond pulse laser (SpectraPhysics-Newport, Santa Clara, CA, USA), with various digital zooms, appropriate emission filters, and excitation at λ_x_^2P^=800 nm, as detailed earlier (Zheng et al., 2015; Mishra et al., 2016). The *z*-stacks of fluorescent images were collected every 5–10 min using 512 × 512 pixel frames (typically 30–50 optical sections in 1-µm *z*-steps) for time-lapse monitoring of Alexa Fluor 488 fluorescence upon microcapsule injection (overall, 5–8 h *in situ*). To provide a direct *in situ* comparison of the diffusion escape rates between non-capsulated and encapsulated Alexa Fluor 488, the former was injected between epidermal and dermal layers with another glass pipette near the microcapsules. In a separate set of experiments, microcapsules carrying Alexa Fluor 488 were placed in glycerol (99%) and monitored using a fast-scanning confocal fluorescence microscope (750–1000 lines/sec) in a photon-count mode, as described.

### Animals in behavioral studies

The animals used for behavioral studies were 2.5- to 3-month-old male Wistar rats. All animal procedures were approved by the local Animal Ethics Committee (Bogomoletz Institute of Physiology, Kyiv, Ukraine): there were performed in full compliance with the ethical guidelines of the International Association for the Study of Pain and the United States Public Health Service’s Policy on Humane Care and Use of Laboratory Animals, as detailed in our previous works (Park et al., 2009b; Kopach et al., 2012, 2016). Animal procedures were fully compliant with the European Commission Directive (86/609/EEC) and the United Kingdom Home Office (Scientific Procedures) Act (1986).

### Experimental design of pain models in vivo

To produce unilateral peripheral inflammation and persistent nociceptive hypersensitivity, 50–100 µl of complete Freund’s adjuvant (CFA; Sigma Chemicals, St. Louis, IL) suspended in an oil:saline (1:1) emulsion was injected subcutaneously into the plantar side of one hind paw of rats (Kopach et al., 2013; Kopach et al., 2015, 2016). Encapsulated QX-314 was injected as a post-treatment of nociceptive hypersensitivity (after induction of the CFA-induced peripheral inflammation). Different groups of animals received a single-intraplantar injection of microcapsules (carrying QX-314 at various concentrations, or empty capsules) into the inflamed tissue of the hind paw, in a total volume of 50 µl (diluted in saline) at 1 day post-CFA, the time point indicating inflammatory pain maintenance (Kopach et al., 2013, 2016). A control group of the CFA-inflamed animals received a similar injection of non-capsulated QX-314 or 2% lidocaine. In a separate set of experiments, groups of non-inflamed animals (no CFA) received encapsulated QX-314 or empty microcapsules. To produce unilateral acute peripheral hypersensitivity of neurogenic origin, 50 µl of capsaicin (1.5 µg/µl, Sigma Chemicals) was injected intradermally into the plantar side of one hind paw.

### Pain assessment

The peripheral thermal pain threshold was measured using the Hargreaves technique, as we described previously (Kopach et al., 2012, 2016). Briefly, after an animal was habituated to a Plexiglas chamber (Biological Research Apparatus, Ugo Basile, Italy), an infrared heat stimulus was applied to the middle of the plantar surface of the hind paw. The heat beam was automatically turned off when the animal lifted its paw. The time between the stimulus onset and the paw withdrawal was automatically recorded: it represented the latency of nociceptive response (thermal pain threshold). Measurements were repeated 3–5 times for each hind paw, the values were averaged.

### Open-field test for explorative behavior and anxiety

To assess the sensorimotor function and animal locomotive behavior, an open-field test (open-field arena) was used as detailed previously (Kopach et al., 2016). In brief, animals were placed in an open-field arena (a 75 × 75 × 40 cm wooden box with a digital camera attached above) and allowed to move freely over a predefined time period (10 min). Recordings were analyzed with MATLAB software using in-house scripts. The following parameters were monitored and recorded to assess locomotion: the total distance traveled by an animal, the maximal speed, and the acceleration. The anxiety level was assessed by computing the ratio between the fractions of time spent in the arena center versus corners.

### Immunohistology

The localization of microcapsules injected *in vivo* was examined with *post hoc* immunohistology of the glabrous skin samples collected from the hind paw at different time points post-injection. The dissected glabrous skin tissue (∼1 cm) was fixed in 3% PFA (1–2 days at 4 °C). After fixation, samples were replaced into PBS containing 0.02% sodium azide and underwent a cryo-protection treatment (10% sucrose for 2 h, then 20% for 2 h, followed by 30% for overnight). Tissue was mounted in OCT and stored at −80 °C until processed for cryo-sectioning. The transverse tissue sections (30–60 μm thick) were cut and collected as an ordered series. Floating tissue sections were rinsed with PBS at least 3 times/5′ each section and blocked with 1% BSA, 5% Donkey serum in 0.2% Triton X-100 PBS for 4 h. The primary antibodies were mouse anti-PGP9.5, neuron cytoplasmic protein 9.5 (1:500; Merck Millipore U.K., Feltham, UK). After incubation with primary antibodies (2 days at 4 °C), tissue sections were incubated with the secondary antibodies (overnight at 4 °C). Secondary antibodies were Alexa Fluor 488 from the same host (1:200 or 1:400; Invitrogen, Paisley, UK). The following-day sections were washed, unrolled and mounted on glass slides using Vectashield H-1400 (Vector Labs, Burlingame, CA, USA). Tissue sections were scanned for both PGP9.5-positive structures and microcapsules using a Radiance 2000 imaging system (Zeiss-Bio-Rad) or Femto2D (Femtonics) imaging system using various digital zooms.

### Microcapsule biodegradability assay

Tissue samples were collected at various time points post-injection (at 1, 5, and 10 weeks), fixed and processed for immunohistology (see above). Special care was taken to make sure that the microcapsule injection parameters (microcapsule concentration in suspension, pressure pulse, etc.) were uniform across the tested samples. To gauge the spatial distribution of microcapsules across the peripheral tissue, we scanned the entire tissue volume (transverse tissue sections cut and collected in a serial order) containing all detectable capsules in a *z*-stack of individual focal *x–y* planes (3-µm *z*-step, total scanned volume of ∼370 μm^3^) using a Femto2D (Femtonics) imaging system. The fluorescence distributed in the *z*-direction thus reported the amount of fluorescently labeled capsules in the tissue volume.

### Statistics

Unless indicated otherwise, summary data are presented as mean ± SEM (standard error of the mean) with *n* referring to the number of cells (*in vitro* experiments) or animals (*in vivo* experiments) in the group. Student’s *t*-test (two-tailed unpaired) was used to determine statistical difference between experimental groups. The statistical difference between groups of experimental animals was analyzed by one-way or two-way analysis of variance (ANOVA) followed by Bonferroni *post hoc* test or Fisher’s test where appropriate. A *p* value of <.05 was considered as statistically significant for either test.

## Results

### Encapsulated QX-314 can gradually suppress neuronal excitability *in vitro*

**Figure 1.**
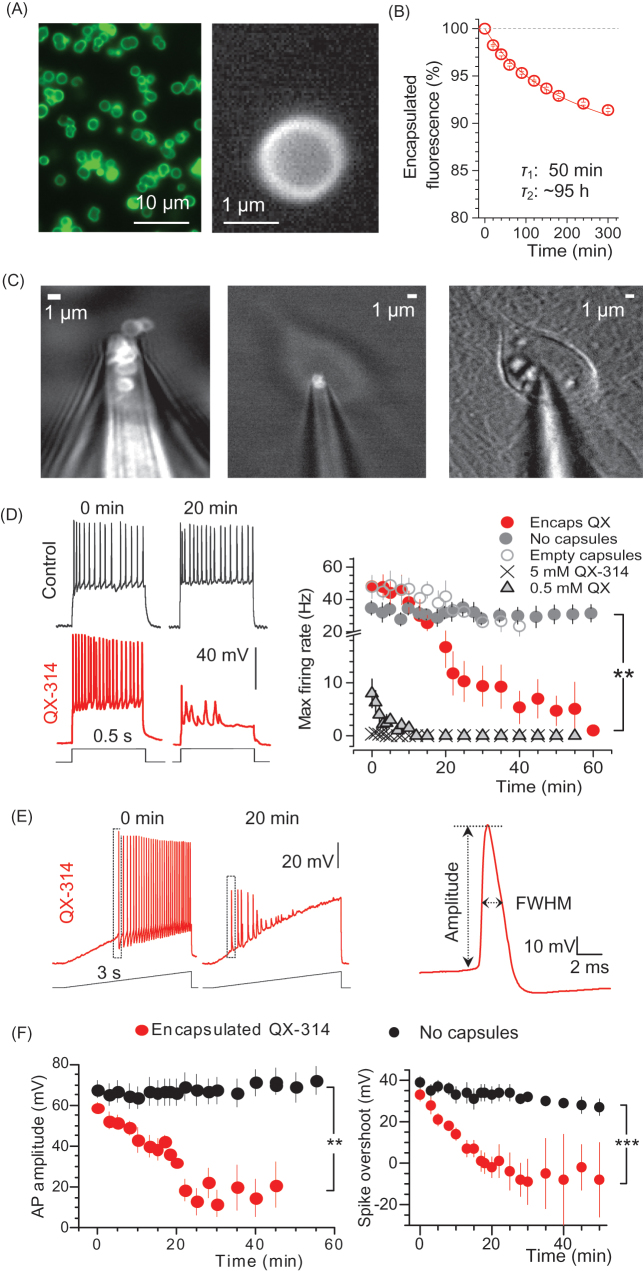
Encapsulated QX-314 delivered intracellularly gradually suppresses neuronal excitability *in vitro*. (A) A snapshot showing the suspension of nano-engineered microcapsules (magnified on the right), with encapsulated Alexa Fluor 488; confocal imaging (λ_x_= 488 nm). (B) Monitoring gradual release of encapsulated Alexa Fluor 488 from microcapsules; ordinate, time course of average capsule fluorescence normalized to the initial value; solid line, best-fit bi-exponential approximation (with 0.04 and 0.96 partial weights, respectively; decay constants as shown; *n* = 1.6 × 10^8^ microcapsules). (C) Snapshots displaying the patch pipette tip filled with microcapsules (TRITC fluorescence) prior to patching (left), when targeting a cultured neuron (middle), and in whole-cell configuration 25 min following intracellular delivery of microcapsules (right; DIC + fluorescence images of the same cell). (D) Traces, examples of current-clamp recordings of neuronal firing in control condition and after injection of encapsulated QX-314 (immediately after breaking-in and 20 min later, as indicated). Graph, statistical summary: time course of the maximal neuronal firing rate in control cells (no microcapsules; *n* = 17), with empty microcapsules injected (*n* = 12), encapsulated QX-314 injected (*n* = 18), with non-capsulated QX-314 injected at 0.5 mM (*n* = 6) and 5 mM (*n* = 4), as indicated. (E) Left, examples of neuronal firing in response to a slow-ramp current after infusion of encapsulated QX-314 (left, 0 and 20 min post-injection in the same cell; dotted boxes indicate first spikes selected for comparisons); right, example of single AP evoked in hippocampal neurons, with the estimated parameters indicated (FWHM, full width at half maximum). (F) Time course of the relative spike amplitude (left) and the spike overshoot (amplitude above 0 mV; right) in control condition (no capsules, black dots) and after intracellular delivery of encapsulated QX-314 (red dots), as indicated; spike sampling as shown in panel E (left); control, *n* = 9 neurons; encapsulated QX-314, *n* = 9 neurons. All data are shown as mean ± SEM. **p < .01, ***p < .001 (unpaired *t*-test).

We fabricated polymeric LbL microcapsules and encapsulated the membrane-impermeable Na^+^-channels blocker QX-314 within the penultimate shell layer (4–10 pg/capsule; Figure 1(A)). The latter was to enable controllable shell permeability for the encapsulated compound. After initial trials, the capsule shell composition was adjusted to provide a cargo release rate of 4–5%/h (decay constant ∼20 h), which was gauged by monitoring the escape of encapsulated fluorescent dye (Alexa Fluor 488, a similar molecular weight to QX-314, Figure 1(B)).

To examine how the encapsulated QX-314 suppresses neuronal excitability, we delivered the microcapsules inside individual hippocampal neurons (primary culture) using a patch pipette in whole-cell configuration (Figure 1(C)). Thus, we monitored cell excitability in real time starting shortly upon microcapsule injection, and compared test groups with control (i.e. cells injected with empty microcapsules, or no capsules, Figure 1(D–F)). We found a gradual suppression of neuronal excitability, which progressed within 15–20 min after injecting encapsulated QX-314, leading to a sharp drop in the maximum firing rate (*n* = 18 cells, *p* < .01 compared with either empty microcapsules, *n* = 12, or no capsules, *n* = 17; Figure 1(D)). In parallel, encapsulated QX reduced the spike amplitude and the spike overshoot (amplitude above 0 mV; *n* = 9, *p* < .001 compared to that with no capsules, *n* = 9; Figure 1(E,F)).

**Figure 2.**
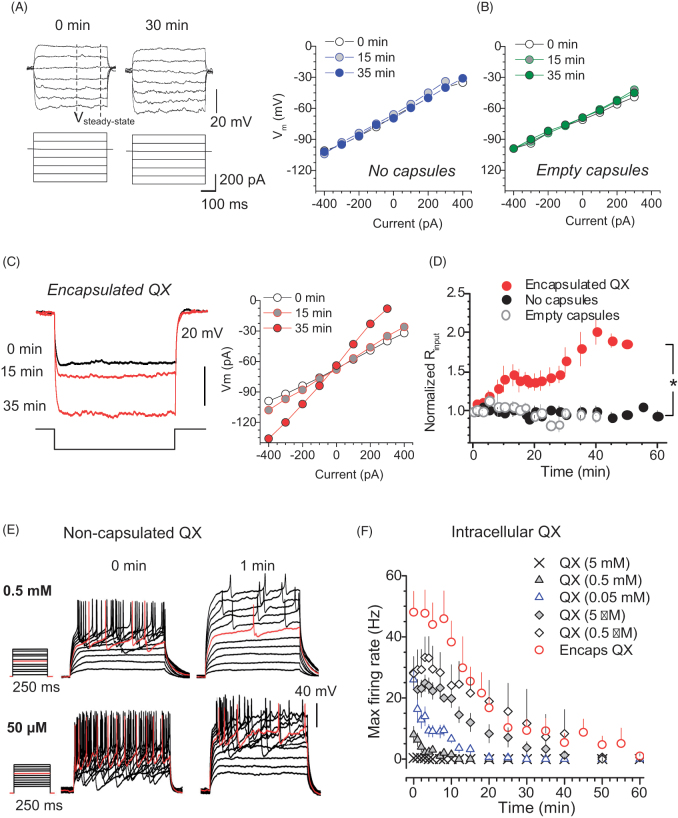
Encapsulated QX-314 gradually increases cell input resistance of neurons while reducing their firing rate in a dose-dependent manner. (A) Traces, examples of the membrane potential response (top) to current step pulses (bottom) in the same neuron (control condition, no microcapsules) at two time points, as indicated. Dashed lines, time window (stable response) where the input resistance was estimated. Graph, examples of the current–voltage relationship at three time points, as indicated, in control conditions. (B) Examples of the current–voltage relationship at three time points, as indicated, after the injection of empty microcapsules. (C) Traces, examples of the membrane potential response (top) to a current injection (bottom) at three time points after intracellular delivery of encapsulated QX-314, as indicated. Graph, examples of the current–voltage relationship at three time points, as indicated, for the same cell. (D) Time course of the cell input resistance in control (*n* = 14 neurons), post-infusion of empty microcapsules (*n* = 15) or encapsulated QX-314 (*n* = 14), as indicated. (E) Traces, representative recordings of neuronal firing after whole-cell dialysis with different concentrations of QX-314, at two time points, as indicated. (F) Time course of the maximum cell firing rate for different concentrations of free QX-314, compared with encapsulated QX-314, added to the intracellular medium, as indicated. Data are mean ± SEM; *p < .05 (unpaired *t*-test).

We also confirmed that the cell input resistance remained stable over time and was not affected by the injection of empty capsules (Figure 2(A,B)). However, it gradually increased following injection of encapsulated QX-314, reflecting major ion channel blockade (*n* = 14, difference at *p* < .05 with the empty-microcapsule group, *n* = 15, or no-capsules group, *n* = 14; Figure 2(C,D)). In contrast, adding QX-314 directly to the intracellular solution (0.5 mM, and especially 5 mM, a commonly used concentration in whole-cell) suppressed spike generation almost immediately (Figure 2(E,F)). To replicate the slow progression of excitability blockade under encapsulated QX-314, intracellular concentration of non-encapsulated QX-314 had to be reduced at least 10^3^–10^4^-fold (to ∼500 nM; Figure 2(F)).

### Microcapsule cargo release rate in skin tissue *in situ*

**Figure 3.**
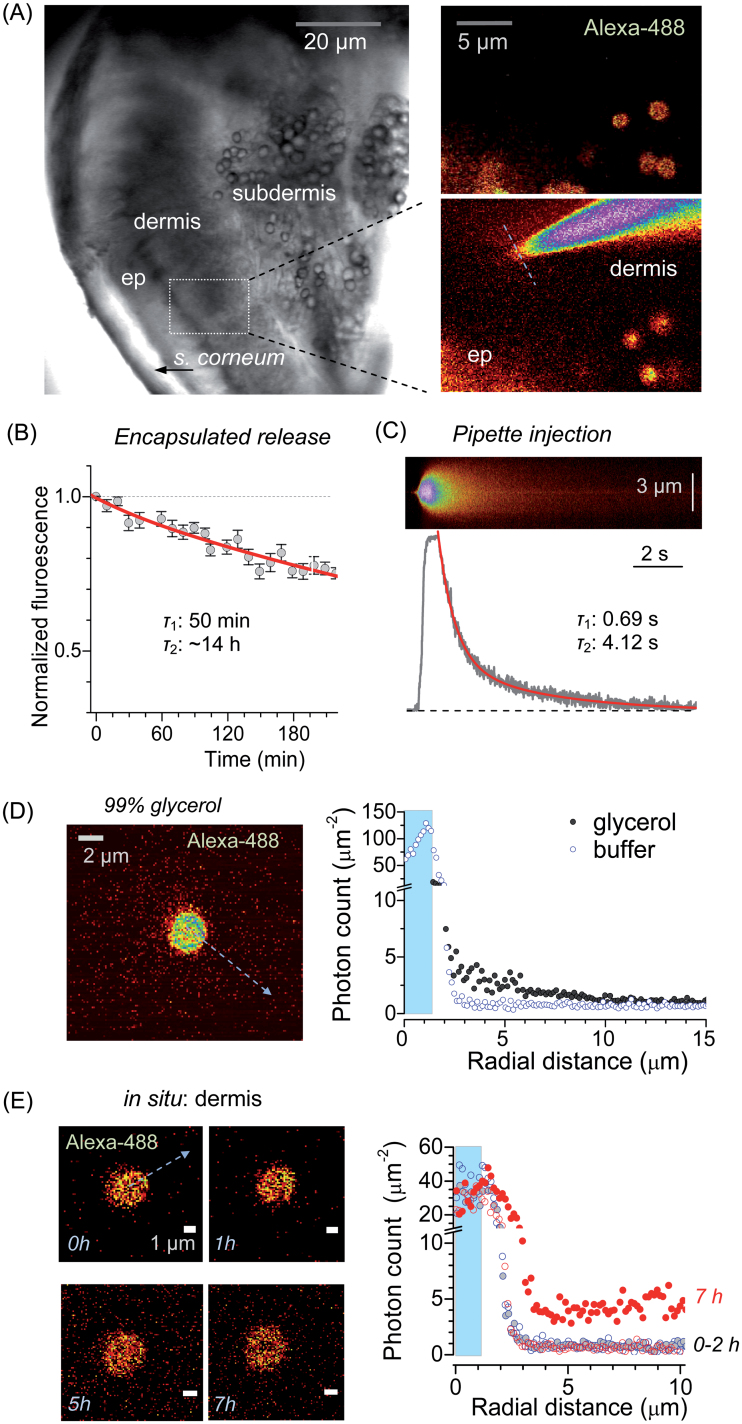
Long-lasting release of encapsulated cargo inside glabrous skin *in situ*. (A) Left image, acute skin tissue preparation from plantar surface of the rat hind paw (DIC image) depicting region of interest (ROI, dotted rectangle). Right images, ROI enlarged and shown in fluorescence channel (λ_x_^2P^ = 800 nm), depicting injected microcapsules (top, encapsulated Alexa Fluor 488), and the subsequently positioned micropipette tip filled with free Alexa Fluor 488 (bottom); dotted line: line-scan position. (B) Concentration kinetics of encapsulated Alexa Fluor 488 release from microcapsules *in situ* (mean ± SEM, *n* = 10); red line, best fit bi-exponential approximation (*τ*_1_, *τ*_2_: decay constants, 0.04 and 0.96 partial weights partial weights, respectively). (C) Image, example of line-scan (position in A, right bottom image; λ_x_^2P^ = 800 nm) depicting the escape of non-capsulated Alexa Fluor 488 injected from the micropipette. Graph, concentration kinetics of free Alexa Fluor 488 injected from the micropipette (0.75 s pressure pulse) *in situ;* red line, best fit bi-exponential approximation (*τ*_1_, *τ*_2_: decay constants, 0.08 and 0.92 partial weights, respectively). (D) Image, experimental arrangement for photon-counting of spatial Alexa Fluor 488 fluorescence escape: a single microcapsule shown in 99% glycerol (arrow, depiction of the radial profile calculation). Graph, radial profiles of Alexa Fluor 488 generated photon counts in two experimental conditions, as indicated (shaded area: capsule radius). (E) Image, example of a microcapsule in the epidermal–dermal area at different time-points (0–7 h, as indicated) following injection into acute skin tissue *in situ* (arrow, depiction of the radial profile calculation). Graph, radial profiles of Alexa Fluor 488 generated photon counts in the epidermal–dermal area, at different time points after injection, as indicated (shaded area: capsule radius).

Because our ultimate goal was to test the pain-relieving effects of encapsulated QX-314, we first sought to establish the microcapsule cargo release properties in peripheral skin tissue *in situ*. To achieve this, we employed an acute skin tissue preparation from glabrous skin of the rat hind paw. Microcapsules with encapsulated Alexa Fluor 488 were delivered through a micropipette between epidermal and dermal layers and monitored using two-photon excitation (2PE) fluorescence microscopy (Figure 3(A)). Microcapsules showed a release rate constant of ∼14 h (slow component), as evaluated by the time-lapse fluorescence emission monitoring of the encapsulated Alexa Fluor 488 *in situ* (Figure 3(B)). In contrast, Alexa Fluor 488 in a free solution injected nearby, directly into the tissue via a micropipette, was escaping at a rate of ∼4.12 s (slow component; Figure 3(C)), i.e. ∼10^4^ times faster. In addition, we used photon counting (within ∼1 µm focal plane), in line with procedures detailed earlier (Zheng et al., 2015), to document a build-up of the diffuse fluorescence profile in the vicinity of individual microcapsules (Figure 3(D)). Within ∼7 h post-injection *in situ*, this profile was consistent with the slow cargo diffusion escape from the microcapsules reported above (Figure 3(E)).

### Encapsulated QX-314 provides long-lasting pain relief *in vivo*

**Figure 4.**
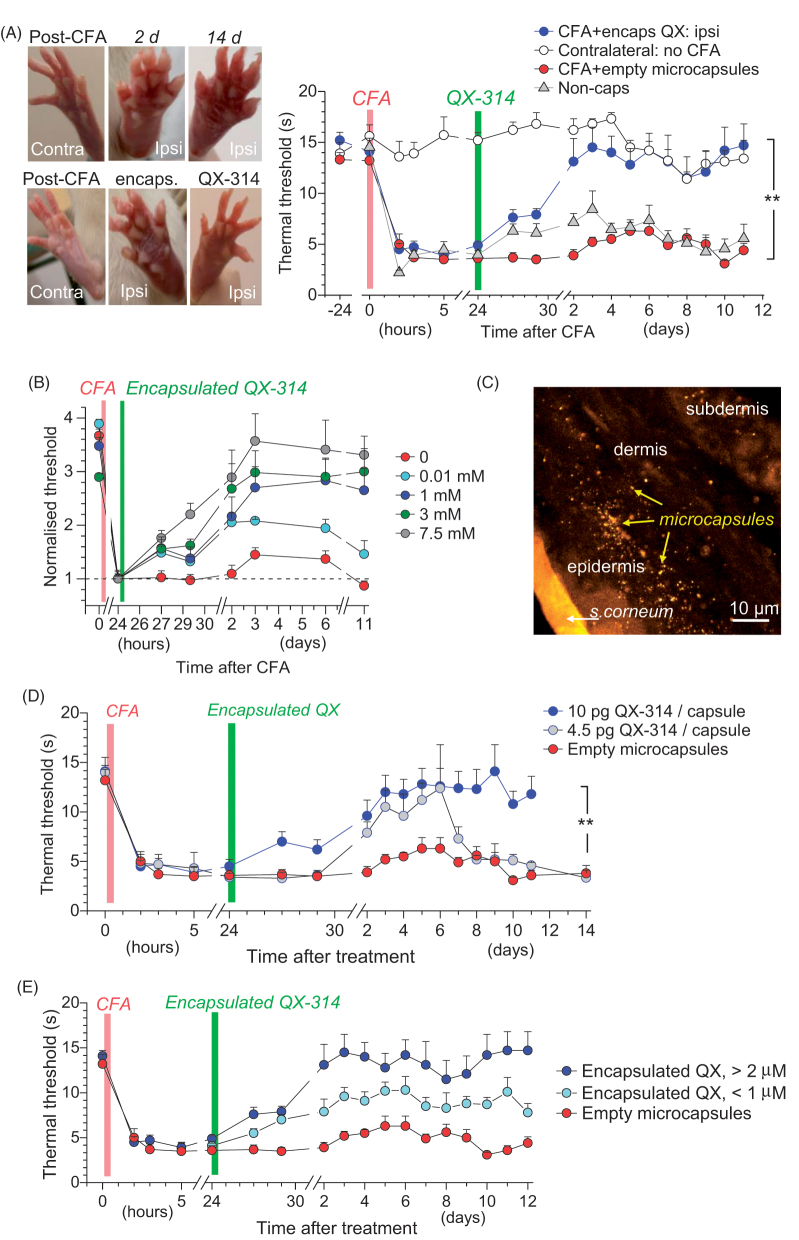
The anti-nociceptive effect of encapsulated QX-314 in a persistent pain model in rodents. (A) Image panels, examples of rat hind paw following injection of CFA with no treatment (top row) and with encapsulated QX-314 (∼3 mM; bottom row) at different time points, as indicated; ipsi, ipsilateral, inflammatory side; contra: contralateral, non-inflamed, side. Graph, time course of the thermal nociceptive threshold in control (contralateral, *n* = 5 rats) and CFA-inflamed animals injected with empty microcapsules (*n* = 5) or encapsulated QX-314 (∼3 mM, *n* = 6) or non-capsulated QX-314 (100 mM, *n* = 5), as indicated. (B) Time course of the thermal nociceptive threshold (normalized to that at the treatment onset, which is 24 h post-CFA), for different dosage of encapsulated QX-314, as indicated: 10 µM (*n* = 6 rats), 1 mM (*n* = 8), 3 mM (*n* = 6), and 7.5 mM (*n* = 7). (C) Fluorescent image (FITC fluorescence, transverse skin tissue section) displaying the epidermal–dermal area (the rat hind paw) with scattered microcapsules on Day 1 after microcapsule injection. (D) Time course of inflammatory hypersensitivity in rats with persistent peripheral inflammation following treatment with different amounts of QX-314 per capsule, as indicated (animals received the same overall dose of QX-314, 1 mM): 4.5 pg (*n* = 6 rats), 10 pg (*n* = 8) and 0 pg (empty, *n* = 5). (E) Time course of inflammatory hypersensitivity with QX-314 in microcapsules of different sizes (animals received the same overall dose of encapsulated QX-314, 3 mM): 2 µm diameter (*n* = 6 rats), 1 µm (*n* = 9) and empty (varied size, *n* = 5). Data are shown as mean ± SEM. **p < .01 (one-way ANOVA with Bonferroni *post hoc* test for encapsulated QX-314 compared with empty microcapsules).

To explore therapeutic potential of encapsulated QX-314, we turned to a well-established experimental paradigm of the CFA-induced unilateral persistent peripheral inflammation (Park et al., 2009b; Kopach et al., 2012, 2016). Following the intraplantar injection of CFA, animals displayed robust thermal hypersensitivity on the ipsilateral (but not contralateral) side, as evaluated by measuring the thermal pain threshold (see ‘Materials and methods’ section). If left untreated, the hypersensitivity persisted for at least 11 days (Figure 4(A)), and a single focal subcutaneous injection of the common analgesic lidocaine had no long-lasting effect (Supplementary Figure S1).

The CFA-inflamed animals were quasi-randomly sampled to receive a single injection of either encapsulated QX-314 or empty microcapsules into the sites of inflammation at 1 day post-CFA. Animals that received encapsulated QX-314 showed substantially alleviated hypersensitivity, with a progressive recovery of the thermal pain threshold back to the pre-inflammatory level by the next day (*n* = 6 rats, *p* < .01 compared with the CFA-inflamed group injected either empty microcapsules, *n* = 5, or no capsules, *n* = 27; Figure 4(A,B). Injecting empty capsules had no detectable effect (*n* = 6 rats, Figure 4(A) and Supplementary Figure S1(A)) whereas a single-focal injection of 100 mM non-encapsulated QX-314 had only a brief and marginal relief of the thermal nociceptive hypersensitivity (*n* = 5 rats, *p* < .05 compared with no treatment at 2 days post-CFA; Figure 4(A)). We could routinely confirm with *post hoc* immunohistology that microcapsules were scattered within the epidermal–dermal area as targeted (Figure 4(C)). Importantly, the therapeutic effect of encapsulated QX-314 remained significant for at least 12 days (*p* < .01 between the CFA-inflamed group injected with 3 mM encapsulated QX and that with empty microcapsules, over the experiment duration; Figure 4(A)) and its time course depended on the concentration of encapsulated QX-314 (Figure 4(B); *p* < .001 between the effects of 7.5 mM encapsulated QX-314, *n* = 7 rats, and empty microcapsules, over 11 days post-CFA; but no difference between 0.01 mM encapsulated QX-314, *n* = 5 and empty microcapsules over the 7–11-day period). Separate experiments showed how the effect depended on the amount of QX-314 encapsulated per capsule (lasting over 12 days post-CFA for microcapsules containing 10 pg of encapsulated QX-314: *n* = 8 rats, *p* < .01 compared with empty microcapsules, but up to 7 days for 4.5 pg of encapsulated QX-314, *n* = 5 rats; Figure 4(D)) and on the capsule size (Figure 4(E); *n* = 6 rats, *p* < .01 for 2 µm microcapsules containing 3 mM of encapsulated QX-314 and *n* = 5 rats, *p* < .05 for 1 µm microcapsules compared with empty ones over the time of experiment).

Control experiments confirmed that in healthy animals (no induced inflammation or pain), neither empty microcapsules (Supplementary Figure 2(A)) nor encapsulated QX-314 (Supplementary Figure 2(B)) affected the peripheral thermal sensitivity, thus ruling out the concomitant effects of microcapsules *per se*. There were no acute (within hours) or delayed (days) effects on thermal sensitivity of the ipsilateral hind paw following intraplantar injection of microcapsules as compared with the contralateral hind paw (no capsules) or with the zero time-point (before injection; Supplementary Figure S2).

### Encapsulated QX-314 rescues locomotive deficit and animal anxiety produced by peripheral inflammation

**Figure 5.**
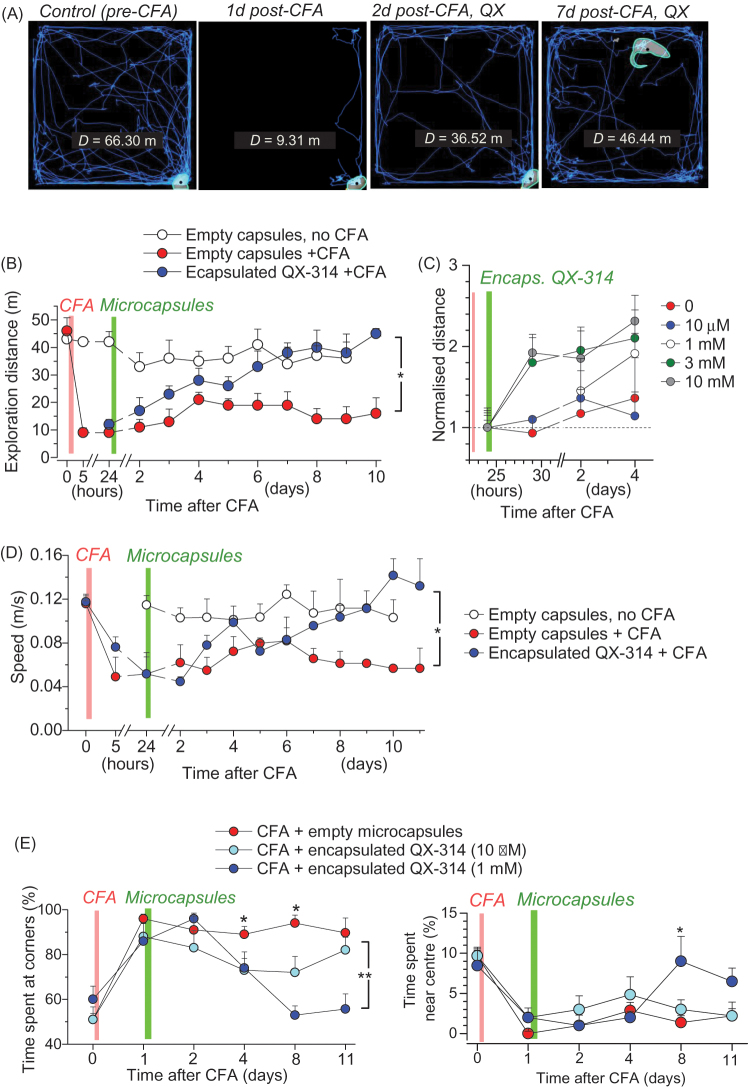
Encapsulated QX-314 abolishes locomotive deficit and anxiety in animals with persistent peripheral inflammation. (A) Examples of the open-field test trajectory records taken from one animal at different time-points following CFA injection and treatment with encapsulated QX-314 (1 mM, 1 day post-CFA), as indicated; *D*, overall distance (m) traveled over 10.0 min. (B) The effect of encapsulated QX-314 on animal locomotion: time course of the average exploration distance traveled by CFA-inflamed animals injected with encapsulated QX-314 (1 mM) or empty microcapsules, and by healthy animals (no CFA) injected with empty microcapsules. (C) The dose-effect of encapsulated QX-314 on exploratory activity in CFA-inflamed animals: time course of the average distance (normalized by that 1 day post-CFA) traveled by CFA-inflamed rats; treatment with empty capsules (*n* = 5 rats), 10 µM encapsulated QX-314 (*n* = 5), 1 mM encapsulated QX-314 (*n* = 5), 3 mM encapsulated QX-314 (*n* = 9), 10 mM encapsulated QX-314 (*n* = 6), as indicated. (D) The effect of encapsulated QX-314 on the average speed that animals could develop following treatment; notations as in (B). (E) The effect of encapsulated QX-314 on the animal’s anxiety, estimated as the average fraction (%) of time spent in the arena corners (correlative anxiety indicator; left) versus the arena center (anti-correlative anxiety indicator; right). Treatment with empty capsules (*n* = 5 rats), 10 µM encapsulated QX-314 (*n* = 5), 1 mM encapsulated QX-314 (*n* = 5). Data are shown as mean ± SEM. *p < .05, **p < .01 (one-way ANOVA with Bonferroni *post hoc* test).

We next examined whether the anti-nociceptive effect of encapsulated QX-314 influences the locomotor deficit and the heightened anxiety, which is often associated with painful states (Kopach et al., 2016). We thus implemented an open-field test, a well-established assay for an integrative analysis of animal behavior, including anxiety (Figure 5(A)). The group of animals with the CFA-induced inflammatory pain showed a dramatic reduction in the traveled distance (*n* = 5 rats, *p* < .01 for each recorded post-CFA time point compared with zero time-point; Figure 5(B)) that persisted for at least 10 days after CFA injection. An integrative analysis of animal behavior demonstrated that encapsulated QX-314 progressively rescued the impaired locomotion in inflamed animals within 2–4 days post-treatment (*n* = 5 rats, *p* < .05 compared with empty microcapsules; Figure 5(B)), in a dose-dependent manner (Figure 5(C)). This was consistent with the improved speed (Figure 5(D)) and acceleration (Supplementary Figure 3(A)). The effect was paralleled by a decline in the anxiety-like behavior, which was monitored as the proportion of time spent near the arena center, as opposed to corners (Figure 5(E)).

### Anti-nociceptive effect of encapsulated QX-314 involves TRPV1 receptors

**Figure 6.**
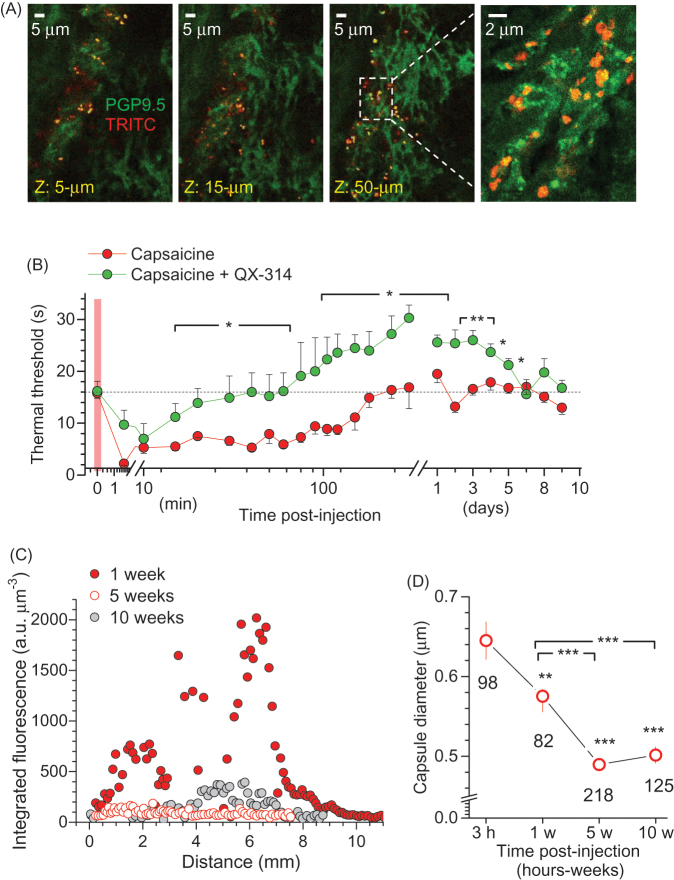
Microcapsules co-localize with peripheral nerve fibers, engage TRPV1-mediated analgesic effect of QX-314, and degrade with a time. (A) Examples of immunohistology of glabrous skin tissue (individual focal planes shown) for microcapsules carrying encapsulated QX-314 (3 mM; TRITC fluorescence, red) surrounding peripheral nerve fibers (neuron-specific PGP9.5 staining, green). Transverse skin sections (30 µm thick). (B) Time course of the thermal pain threshold in animals injected with capsaicin (1.5 µg µl^−1^, *n* = 6 rats) or with capsaicin together with 7.4 mM QX-314 (non-capsulated, *n* = 6). Data are mean ± SEM. *p < .05, **p < .01 (one-way ANOVA with Bonferroni *post hoc* test). (C) The *z*-axis profile of the microcapsule fluorescence (TRITC, λ_x_^2P^ = 820 nm; 3 mM encapsulated QX-314, integrated fluorescence) inside the skin tissue from the rat hind paw at different times post-injection *in vivo*, as indicated. Total scanned volume ∼370 µm^3^; 3 µm *z*-steps. (D) Averaged diameter of microcapsules inside the skin tissue from the rat hind paw at different time points post-injection *in vivo*, as indicated. The reduced diameter reflects progressive degradation of the outer capsule shell. Total number of analyzed capsules is indicated; **p < .01, ***p < .001 (one-way ANOVA with Fisher’s test).

Because QX-314 acts on Na^+^ channels from the intracellular side, it has to be transported into nerve fibers once released inside the tissue. The key established mechanism of this permeation involves TRP-dependent receptors TRPV1 (Binshtok et al., 2007; Peters et al., 2014) and TRPA1 (Roberson et al., 2011; Brenneis et al., 2014), although the Toll-like receptor-5 could also contribute (Xu et al., 2015). First, we confirmed that the injected microcapsules rest near the peripheral nerve fibers (identified with neuron-specific PGP9.5 staining) innervating skin tissue layers (Figure 6(A)). To determine whether the QX-314 action involves the TRP-mediated pathway, we next used a model of neurogenic pain induced by capsaicin, a prototypic TRPV1 agonist (Frias & Merighi, 2016).

Capsaicin (1.5 µg/µl) produced thermal hyperalgesia within a minute (*n* = 6 rats, *p* < .01), with an immediate adverse reaction of the animal (licking, shaking the injected hind paw); the thermal hypersensitivity lasted up to 2–3 h, reversing back to control values (Figure 6(B)). QX-314 (7.4 mM, non-capsulated) co-injected with capsaicin rapidly produced anesthesia, which gradually developed into analgesia to a heat stimulus within 3–5 h post-injection (*n* = 6 rats, *p* < .05 compared with capsaicin at the corresponding time-points), fully consistent with previous observations (Binshtok et al., 2007). The anti-nociceptive effect of QX-314 remained for several days (*n* = 6 rats, *p* < .05 compared with capsaicin at 1–5 days post-injection; Figure 6(B)). In contrast to the complex cellular signaling mechanisms mediating the CFA-induced persistent inflammatory pain, the capsaicin-induced neurogenic pain should specifically involve TRPV1 activation at primary nociceptor afferents. Therefore, the most parsimonious explanation of the prolonged analgesic effect observed in this study is that QX-314, once injected subcutaneously, gets into the primary nociceptors through the activated TRPV1 receptors and retained inside nerve fibers.

### Microcapsules undergo progressive biodegradation post-injection *in vivo*

The progressive and nontoxic biodegradation of the fabricated microcapsules is important for their safe biomedical use. The capsules of a similar design and polymer composition have already been validated for biodegradability and decomposition in human cell lines (De Cock et al., 2010; Pavlov et al., 2011; Gao et al., 2016). The constituent nanomaterials have also been tested for their low-toxicity biodegradation *in vivo* (Park et al., 2009a; Chiappini et al., 2015). To confirm the satisfactory biodegradation of microcapsules used here, a *post hoc* immunohistology was carried out in tissue samples collected at different time-points after injecting microcapsules *in vivo*.

We assembled the integrated microcapsule fluorescence distributed in the *z*-direction to gauge the amount of fluorescently labeled capsules in the tissue volume (total scanned volume of ∼370 μm^3^). Five weeks or later post-injection, this distribution was reduced dramatically (Figure 6(C); there was no detectable photo-bleaching of TRITC in individual remaining capsules at these time-points). In parallel, we monitored the average diameter of the remaining microcapsules (those with a distinguishable shape). As biodegradation of the multiple layers comprising the capsule shell is expected to start from the outer layer, it should gradually reduce the visible capsule size—this we precisely observed (*p* < .01 or *p* < .001 compared with the initial time-point, 3 h post-injection; Figure 6(D)) as a confirmation of robust enzymatic decomposition of the microcapsule shell material within live tissue over a time post-injection.

## Discussion

Improving the treatment of persistent pain is amongst the most challenging tasks in clinical practice today, directly affecting the lives of millions. Systemic administration of established painkillers over a prolonged period of time tends to have serious side effects and is therefore considered suboptimal. When the peripheral source of pain can be localized, the existing methods of local anesthesia rely either on a direct repetitive drug injection or on the permanent drug-administering devices. Both methods have serious limitations pertinent to the patient’s discomfort, the costs involved, and a possible systemic escape of the drug.

The present study was prompted by the recent breakthroughs in the nano-engineering LbL technology, which enables micro-encapsulation and subsequent controllable release for the medicine of choice. A wide variety of substances have successfully been used as a payload for encapsulation (De Cock et al., 2010). The capsule size could be adjusted between 0.3 µm and >10 μm (determined by their template), whilst the surface functionality is provided by the outer layers. Crucially, the capsule shell permeability can be made sensitive to environmental variations (pH, temperature, osmolarity) (Delcea et al., 2011) or a remote physical stimulus (light, magnetic field, ultrasound) (Antipina & Sukhorukov, 2011), which is achieved through the incorporation of sensitive elements into the multilayer capsule wall (Munoz Javier et al., 2008; Pavlov et al., 2011, 2013; Gao et al., 2016). The LbL microcapsules normally show no toxicity or appreciable inflammation effects (Pavlov et al., 2011) and in most cases they are fabricated using standard biodegradable components (e.g. hyaluronic acid, dextran derivatives and other polysaccharides, gelatin and other peptides) approved by the FDA and equivalent European regulatory authorities for use in humans (Zhang et al., 2013). They therefore generally meet the requirements for a safe drug delivery platform suitable for use in humans.

In parallel, there has been a growing interest in the Na^+^-channel blocker QX-314, which appears to be more potent and longer-lasting in its analgesic effects in mammals, compared to the more traditional painkillers (Lim et al., 2007; Roberson et al., 2011; Zhao et al., 2014). It is also routinely used in experimental neurophysiology research for dampening neuronal excitability. Although QX-314 is a membrane-impermeable compound which blocks Na^+^ channels from the cytoplasm side, studies *in situ* have established that it enters nerve cells through the TRP receptors subtypes TRPV1 and TRPA1 (Binshtok et al., 2007; Leffler et al., 2011; Stueber et al., 2016) and possibly also through a TRP-independent mechanism (Brenneis et al., 2014; Hofmann et al., 2014).

Our research strategy sought to combine these two recent lines of enquiry, aiming to test it in a case study in rats whether encapsulated QX-314 could improve peripheral pain treatment compared to more traditional approaches. This strategy achieved several objectives. Firstly, we nano-engineered encapsulated QX-314, injected the microcapsules into individual nerve cells (in culture) and documented gradual suppression of excitability in individual neurons under whole-cell electrophysiological control. Secondly, we evaluated and adjusted the microcapsule properties in the target tissue areas *in situ* to ensure relatively slow, long-term local release of the encapsulated cargo. Thirdly, and perhaps most importantly, we found that a single local injection of encapsulated QX-314 had a robust anti-nociceptive effect in an animal model of persistent inflammatory pain *in vivo*. The effect, which lasted for more than 1 week, was documented using the semi-automated measurements of the pain threshold and camera-traced quantification of the animal locomotion and explorative behavior. The results were gauged against the groups of inflamed animals which received a single injection of either empty microcapsules or non-encapsulated QX-314, and also the control groups of non-inflamed animals or those with an injection of empty microcapsules. Finally, we established that, consistent with the previous observations, the prevalent cellular mechanism enabling intracellular QX-314 action is likely to involve TRPV1 receptors and that the microcapsules undergo robust biodegradation from Week 5 post-injection onwards.

Clearly, although the LbL microcapsules are proving themselves as a highly flexible method of drug encapsulation and delivery, the injection method described here provides relatively limited control over their precise positioning, dosage, and spread in the target tissue. Remote control of microcapsule movement inside tissue could further improve the coordinate precision of drug delivery. Another promising objective for the future development is to equip the surface of microcapsules with physicochemical properties that would help to recognize target tissue or cells. In terms of practical application, further tests are needed to establish the stability and the storage requirements for the microencapsulated medicine, including the costs involved. Nonetheless, the present results suggest that LbL microcapsules represent a promising approach to provide long-lasting, efficient and controllable focal delivery of anti-nociceptive drugs, with limited side effects on the systemic level.

## Supplementary Material

Olga_et_al._Supplementary_Material.pdf
