# Supplementary material for: Nano-engineered microcapsules boost the treatment of persistent pain
Source: Drug Deliv. 2018 Jan 31;25(1):435–47. doi: 10.1080/10717544.2018.1431981 (PMC5796488; doi:10.1080/10717544.2018.1431981)
Supplement: Olga_et_al._Supplementary_Material.pdf [file IDRD_A_1431981_SM3307.pdf]

Nano-engineered microcapsules boost the treatment of persistent pain

Additional Figures

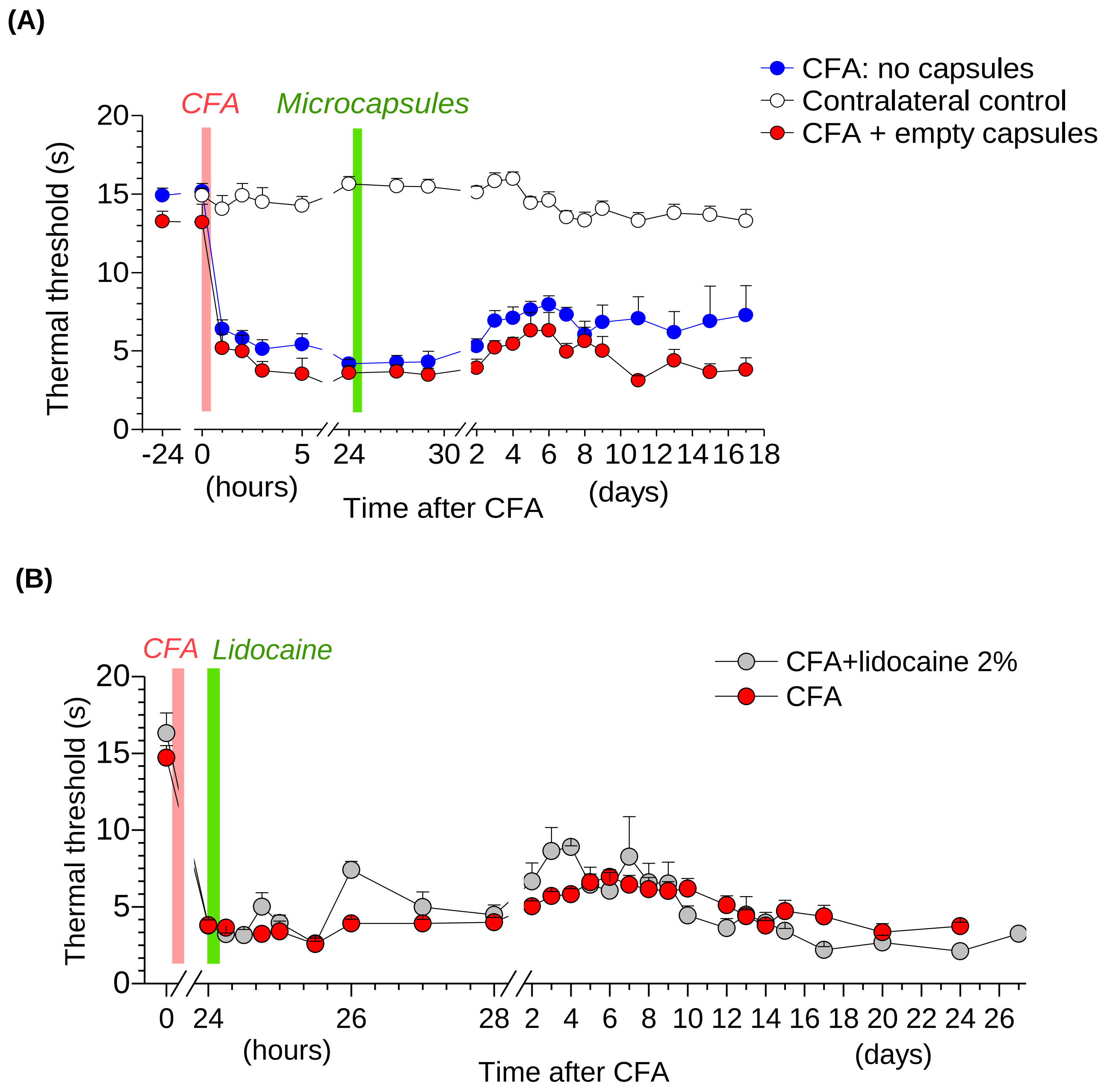

Additional Figure 1. Persistent inflammatory pain remains refractory to single focal injection of lidocaine.

(A) Time course of the thermal nociceptive threshold (Hargreaves test) for the CFA-inflamed animals. Empty microcapsules (no payload) produced no changes in the CFA-induced peripheral thermal hypersensitivity in rats after injection into inflamed tissue, as indicated. Number of animals tested: n = 27 with CFA-induced peripheral inflammation, no capsules, and n = 6 CFA-inflamed rats injected with empty microcapsules.

(B) Time course of the thermal nociceptive threshold in CFA-inflamed animals with no treatment or injected with 2%, as indicated; n = 15 rats for CFA group, n = 5 CFA-inflamed rats treated with lidocaine.

Data are shown as mean ± SEM.

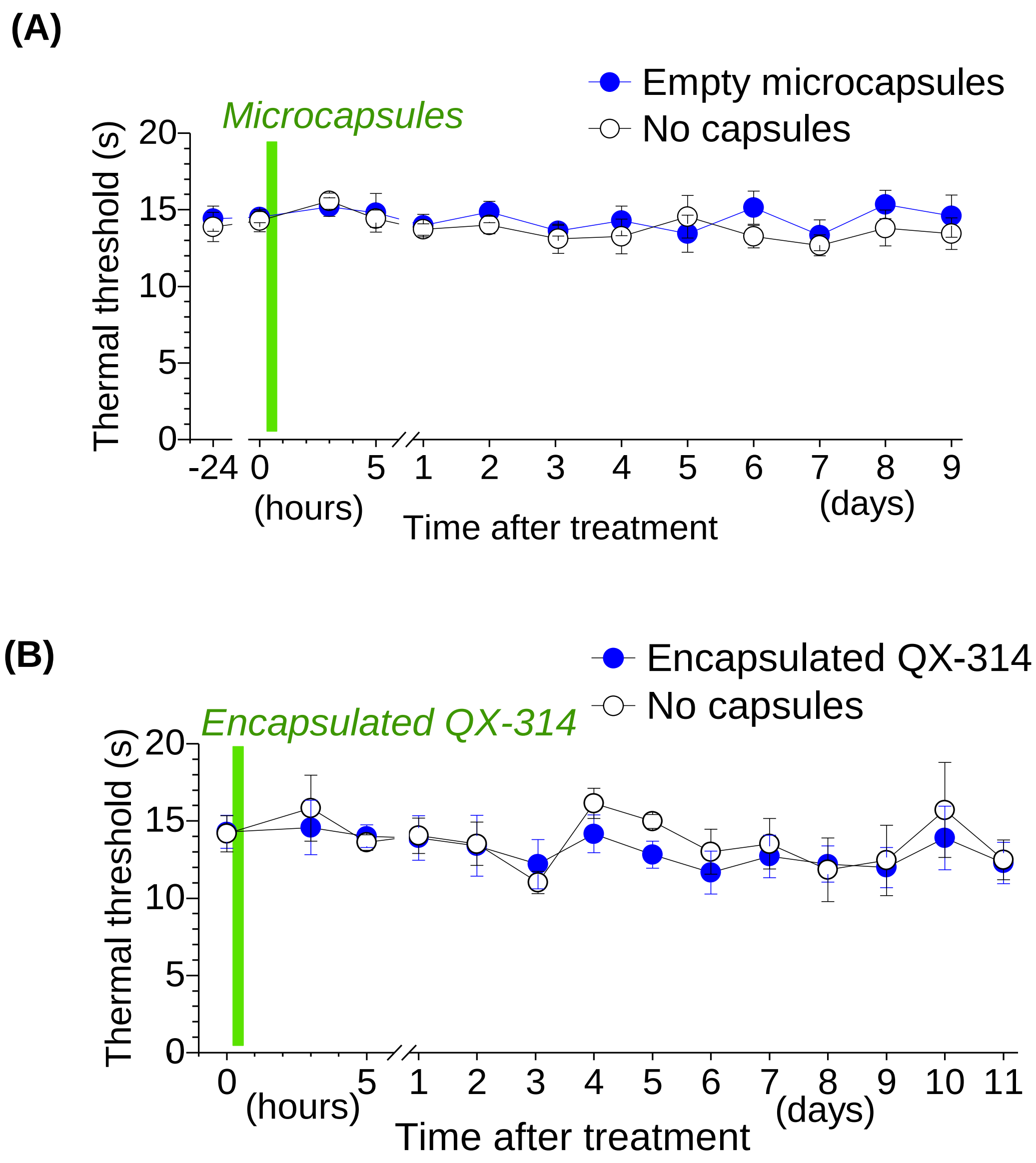

Additional Figure 2. Testing the concomitant effects of microcapsules *in vivo*.

In non-inflamed animals, empty microcapsules (no payload, **A**) or encapsulated QX-314 (**B**) had no effect on the time course of the thermal threshold of the ipsilateral hind paw compared to naïve animals (no microcapsules injected). Number of animals tested: n = 5 rats for empty microcapsules, n = 6 animals for encapsulated QX-314. Data are mean ± SEM.

(A)

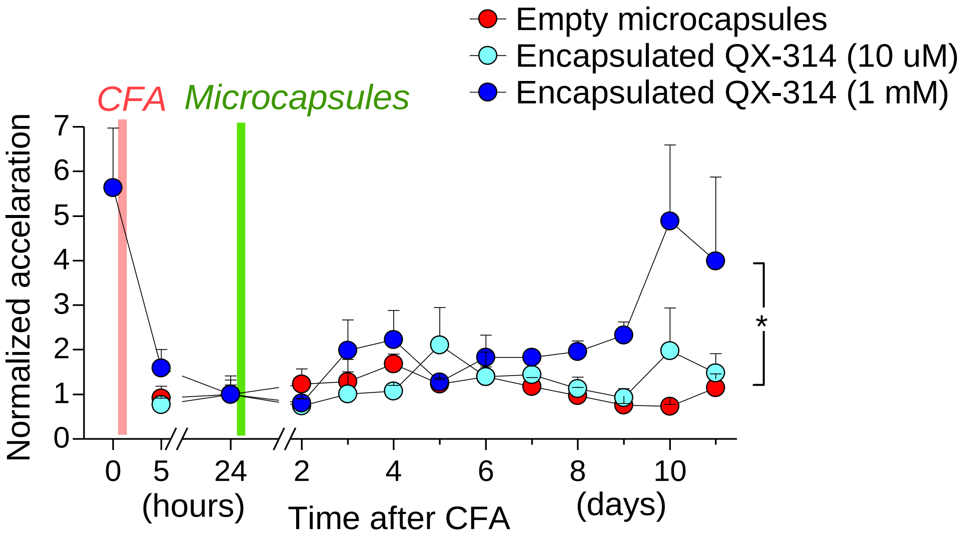

(B)

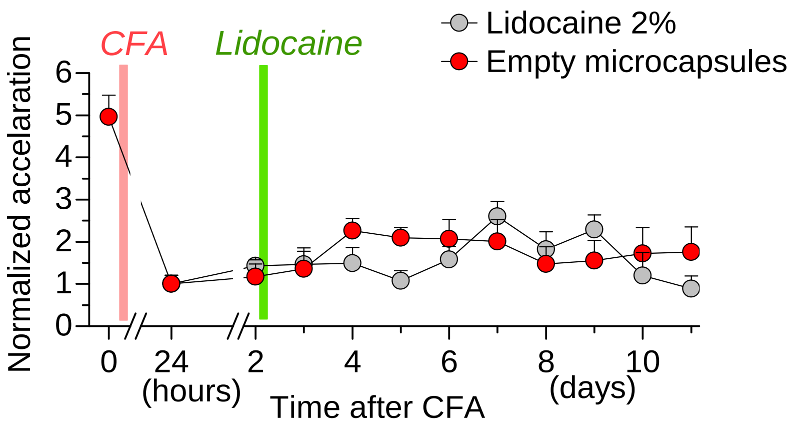

Additional Figure 3. Encapsulated QX-314 improves impaired locomotion in animals with persistent peripheral inflammation.

(A) The dose-dependent effect of encapsulated QX-314 on the average acceleration that animals with inflamed hind paw display in an open-field test following treatment, as indicated. Number of animals tested: n = 5 rats for

empty microcapsules, n = 4 animals for 1 mM encapsulated QX-314; n = 5 animals for 10 μM encapsulated encapsulated QX-314.

(B) Time course of the average acceleration that animals with the CFA-inflamed hind paw display in an open-field test following lidocaine treatment (a single injection of 2% lidocaine) or injection of empty microcapsules, as indicated. Number of animals tested: n = 6 treated with lidocaine, n = 5 injected with empty microcapsules.

Data are shown as mean ± SEM. * $p < 0.05$ (one-way ANOVA with Bonferroni post hoc test).
